# Fungal infestation boosts fruit aroma and fruit removal by mammals and birds

**DOI:** 10.1038/s41598-017-05643-z

**Published:** 2017-07-17

**Authors:** Josep E. Peris, Ana Rodríguez, Leandro Peña, José María Fedriani

**Affiliations:** 1 Laboratorio de Biotecnologia Vegetal. Pesquisa & Desenvolvimento. Fundo de Defesa da Citricultura (Fundecitrus). Vila Melhado, 14807-040 Araraquara, São Paulo Brazil; 2 Instituto de Biología Molecular y Celular de Plantas (IBMCP). Consejo Superior de Investigaciones Científicas (CSIC)-Universidad Politécnica de Valencia (UPV), 46022 Valencia, Spain; 3 Centro de Ecologia Aplicada “Prof. Baeta Neves”/InBIO. Instituto Superior de Agronomia. Universidade de Lisboa, 1349-017 Lisboa, Portugal; 4 Estación Biológica de Doñana (CSIC). Isla de la Cartuja, 41092 Sevilla, Spain

## Abstract

For four decades, an influential hypothesis has posited that competition for food resources between microbes and vertebrates selects for microbes to alter these resources in ways that make them unpalatable to vertebrates. We chose an understudied cross kingdom interaction to experimentally evaluate the effect of fruit infection by fungi on both vertebrate (mammals and birds) fruit preferences and on ecologically relevant fruit traits (volatile compounds, toughness, etc). Our well-replicated field experiments revealed that, in contrast to previous studies, frugivorous mammals and birds consistently preferred infested over intact fruits. This was concordant with the higher level of attractive volatiles (esters, ethanol) in infested fruits. This investigation suggests that vertebrate frugivores, fleshy-fruited plants, and microbes form a tripartite interaction in which each part could interact positively with the other two (e.g. both orange seeds and fungal spores are likely dispersed by mammals). Such a mutualistic view of these complex interactions is opposed to the generalized idea of competition between frugivorous vertebrates and microorganisms. Thus, this research provides a new perspective on the widely accepted plant evolutionary dilemma to make fruits attractive to mutualistic frugivores while unattractive to presumed antagonistic microbes that constrain seed dispersal.

## Introduction

Human activity creates numerous opportunities for the appearance of so-called ‘novel interactions’^1^ between species that otherwise would not coexist. Novel interactions arising from biological invasions have been deeply investigated^2^ and pair-wise interactions between native and crop species have also received some attention^3–5^. Surprisingly, novel cross-kingdom (e.g. plant-vertebrate-microbe) interactions remain largely understudied despite their pervasiveness and potential ecological, evolutionary, and economical relevance in natural and humanized ecosystems^2, 6^. In particular, novel cross-kingdom interactions taking place in agro-ecosystems provide excellent logistical settings to investigate, through easily replicated field experiments, intriguing ecological and evolutionary questions.

Interactions between fruiting groves and frugivorous vertebrates are widespread worldwide^7–9^. Seeds of domestic species are often ingested by vertebrates and eventually released away from the mother plant (i.e. endozoochory), potentially leading to naturalization of such cultivated plants^10^. Interestingly, these bipartite interactions can be joined by microorganisms that feed on the pulp and seeds, thus potentially interfering with the domestic plant-seed disperser interaction. Janzen^11^ suggested that fruits infested by microbes are rarely eaten by vertebrates because microbes produce toxic compounds and reduce the nutritional value of infested fruit. Most empirical investigations of these cross-kingdom interactions^12–14^ as well as recent theoretical evidence have supported Janzen’s famous prediction^15^, but see^16^. Nonetheless, there exist a handful of studies showing that frugivores prefer infested fruits^9, 17–19^. Also, recent paleogenetic data suggest that during the middle Miocene (~16 MA ago), apes evolved the ability of ingesting fallen microbe-infested fruit^20^. Such disparate findings are potentially related to frugivore response to fruit infestation changing with vertebrate, plant, and/or pathogen species, as well as with the ecological context^14, 21^. Surprisingly, however, most available empirical evidence is restricted to interactions involving small fruits and small frugivorous birds (see Supplementary Table S1), and extensively replicated field experiments assessing the spatial and temporal consistency of frugivore responses to microbe infestation are lacking. Furthermore, whether different frugivore functional groups with contrasting foraging modes (e.g. seed disperses, pulp feeders, seed predators^22, 23^) respond similarly to microbe infestation is unknown. Though species involved in novel interactions do not share a common evolutionary history, these tripartite interactions could shed light on how plants solve the evolutionary dilemma of making their fruits attractive to seed dispersers while unattractive to antagonistic microbes^13, 15^.

We also know very little concerning the mechanisms underlying vertebrate responses to microbe fruit infestation. In particular, plant volatile organic compounds (VOCs) are secondary metabolites that mediate the attraction of seed dispersers and the avoidance of seed predators^24, 25^. VOCs emitted by fruits and altered by microorganism infestation are known in some domestic species^26, 27^. Whether these microbe-induced VOCs changes together with other potential changes in fruit physical and chemical proprieties (e.g. toughness, sweetness, pH) have a significant effect on subsequent fruit interactions with native vertebrate frugivores remains a puzzle^19^.

Frugivorous vertebrates, domestic *Citrus* trees, and microorganisms form widespread though understudied tripartite interactions^9, 27, 28^. In particular, *Penicillium digitatum* Sacc. infects large fractions of harvested orange fruits worldwide^27^. In this study, we chose the interaction among the sweet orange tree (*Citrus sinensis* L. Osb.), several mammalian and avian frugivores, and *P. digitatum* in tropical and Mediterranean groves to experimentally address the following four questions: *i*) Does infection by *Penicillium* alter orange fruit preferences by different vertebrate frugivores (mammals, birds)? If so, and given their contrasting frugivore faunas, *ii*) is such effect consistent between tropical and Mediterranean orange groves? Also, since different frugivore guilds (i.e. seed dispersers, pulp feeders, granivore rodents) often show contrasting foraging behaviors^23, 29^, *iii*) is the effect of fungal infestation consistent among frugivore guilds? Finally, *iv*) does *Penicillium* infestation alter physical and/or chemical orange fruit parameters (e.g. toughness, VOC profiles, acid/sugar and ethanol concentrations) and, if so, are such changes consistent with frugivore fruit preferences?

## Results

### Effect of Penicillium infestation on fruit harvesting

Frugivore tracks and/or other signs such as feces were found by the fruit in all experimental orange trees both in the Mediterranean and the tropical experimental groves. Whereas in the Mediterranean groves, we recorded visits by pulp feeders (mostly rabbits) and rodents, in the tropical groves, in addition to those two frugivore guilds, we also recorded frequent visits by seed dispersers (mostly wild boars; see Supplementary Methods and Results).

**Figure 1:**
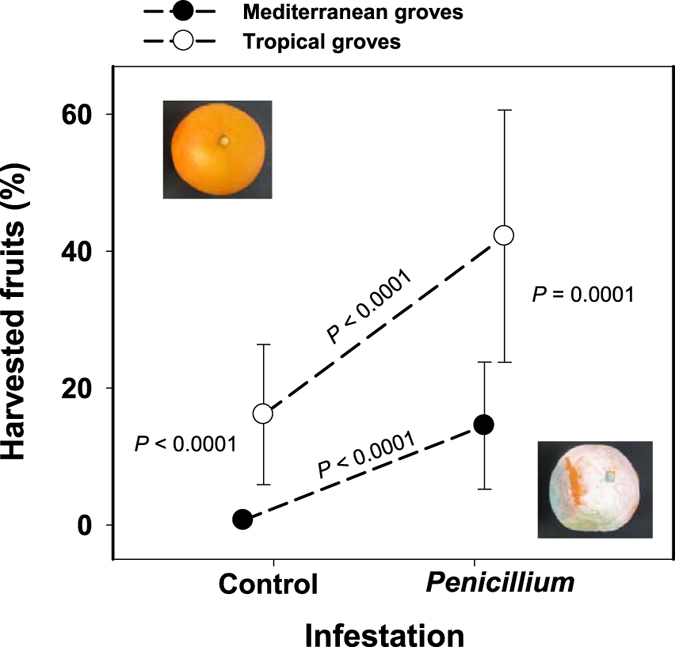
Harvesting by vertebrate frugivores (mammals and birds) of intact and *Penicillium*-infested oranges. Graphical representation of statistically significant interaction between *Penicillium* infestation (intact *vs*. *Penicillium*-infested) and region (tropical *vs*. Mediterranean) found for overall fruit harvesting by vertebrate frugivores of sweet orange (*Citrus sinensis*) fruit in our experimental Mediterranean and tropical groves. The *P*-values of the tests for the four simple main effects involved in the interaction are shown.

**Figure 2:**
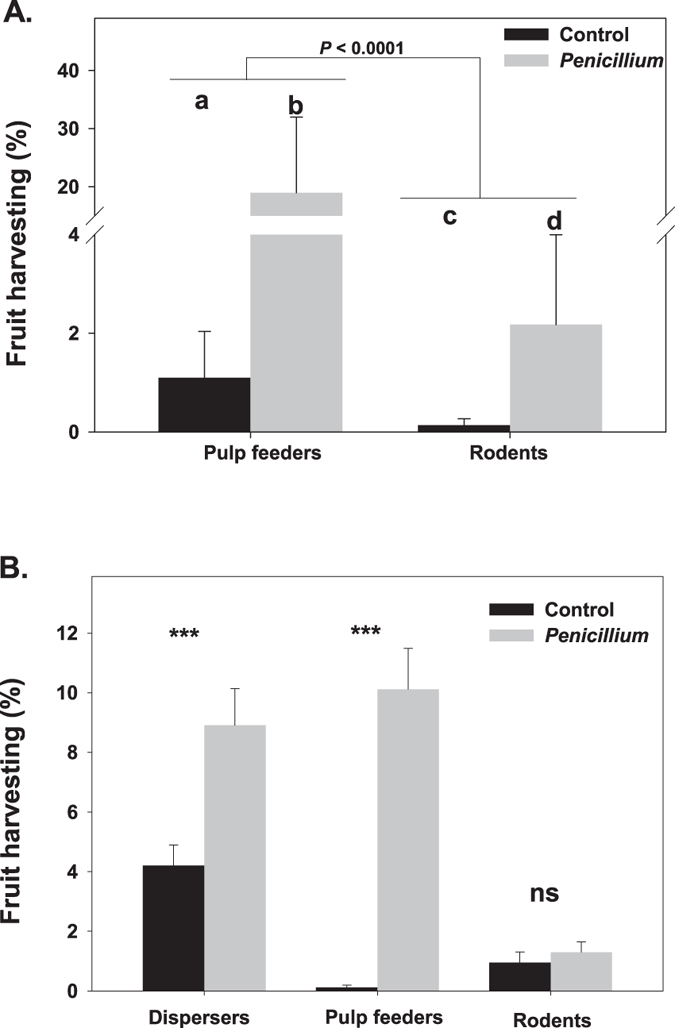
Fruit harvest by different frugivore guilds (i.e. seed dispersers, pulp feeders, granivore rodents). Model corrected mean percentages (±1SE) of sweet orange (*Citrus sinensis*) fruit harvest by different frugivore guilds as a function of *Penicillium* infestation in the Mediterranean (**A**) and tropical groves (**B**). Different lowercase letters among *Penicillium* infestation levels denote significant (*P* < 0.05) differences. ***P < 0.0001; ns, not significant (*P* > 0.05).

Our mixed model indicated that, once the effects of random factors were controlled for, overall fruit harvesting was 8.2-fold higher in the tropical than in the Mediterranean groves (F_1, 2897_ = 118.90, *P* < 0.0001; Fig. 1). Frugivores from both regions strongly preferred *Penicillium*-infested as compared with intact fruits (F_1, 2897_ = 551.31, *P* < 0.0001; Fig. 1). Nevertheless, there was a significant interaction between *Penicillium* infestation and region (F_1, 2896_ = 93.65, *P* < 0.0001) indicating that the effect of infestation on fruit harvesting was stronger in the Mediterranean orange groves as compared with the tropical ones (Fig. 1). Specifically, in the Mediterranean groves, harvesting percentage for *Penicillium*-infested fruit was 21.1-fold higher than for intact fruit (tests of slices, F_1, 2896_ = 369.01, *P* < 0.0001), whereas in Brazil fruit harvesting percentage for *Penicillium*-infested fruit was 2.6-fold higher than for intact fruit (F_1, 2897_ = 187.42, *P* < 0.0001). We undertook a second set of analyses to identify guild-specific effects of *Penicillium*-infestation on fruit harvesting in each region (Table 1). In the Mediterranean groves, there were strong overall significant differences between guilds in the percentages of fruit harvesting (*P* < 0.0001; Table 1), with pulp feeders harvesting 8.9-times more fruits than rodents (Fig. 2A). Also, we found a strong significant *Penicillium* infestation effect on fruit harvesting, being 17.23-times higher for infested as compared to intact orange fruits (Table 1; Fig. 2A). The non-significant interaction between infestation and guild (Table 1) indicated that the marked preference for infested fruits was consistent on sign and magnitude for both frugivore guilds (Fig. 2A).

**Table 1:** Results of main effect tests using generalized linear mixed models on the effects of *Penicillium digitatum* infestation (P) and consumer guild (G), as well as their second-order interaction, on percentages of fruit harvesting in Mediterranean and tropical sweet orange (*Citrus sinensis*) groves.

	Mediterranean groves			Tropical groves		
	F	d.f.	*P*	F	d.f.	*P*
*Penicillium* (P)	138.25	1, 3062	**<0.0001**	63.64	1, 4215	**<0.0001**
Guild (G)	80.30	1, 3062	**<0.0001**	49.20	2, 4215	**<0.0001**
P *G	0.29	1, 3062	0.561	32.51	2, 4215	**<0.0001**

In the tropical groves, seed dispersers (6.70 ± 0.88%) harvested significantly more fruits than both seed-eating rodents (1.09 ± 0.27%) and pulp feeders (4.87 ± 0.68%; Table 1). As in the Mediterranean groves, overall fruit harvesting was significantly (Table 1) higher for *Penicillium*-infested as compared with intact fruit (Fig. 2B). Interestingly, however, there was a strongly significant interaction between guild and *Penicillium*-infestation (Table 1) indicating that the magnitude and/or direction of *Penicillium* infestation effect on fruit harvesting varied among frugivore guilds (Fig. 2B). In particular, test of slices showed no significant *Penicillium* infestation effect on rodent fruit harvesting (F_1, 4215_ = 1.10, *P* = 0.294), whereas seed dispersers (F_1, 4215_ = 38.32, *P* < 0.0001) and, particularly, pulp feeders (F_1, 4215_ = 60.54, *P* < 0.0001) markedly selected infested fruit (Fig. 2B). Overall, our results indicated a strong vertebrate frugivore preference for *Penicillium*-infested fruits. Furthermore, such fruit preference took place in frugivores as contrasting as wild boars, ring-tailed coatis, curl-crested jays and ruddy ground doves in the tropical groves, or European rabbits, field mice, blackbirds, and magpies in the Mediterranean groves.

### Effect of *Penicillium* infestation on VOC emission and on fruit physical and chemical traits

Our VOC analyses of intact and infested oranges identified 55 and 89 different compounds in intact and *Penicillium*-infested fruits, respectively (the entire list can be found as Supplementary Table S4). Both types of fruits differed significantly in mean percentages of volatile compounds, both for all compound groups considered individually (i.e. GLM univariate tests) and when all volatiles were treated simultaneously in a multivariate analysis of variance (MANOVA, F_5, 8_ = 63.23, *P* < 0.0001). For instance, percentage of esters in infested fruits was 5.7-fold higher as compared with intact fruits (F_1_ = 169.88, *P* < 0.0001), whereas percentages of hydrocarbons was 1.3-fold higher in intact as compared to infested fruits (F_1_ = 249.6, *P* < 0.0001; Fig. 1). The differences between fruit types in percentages of alcohols was small but significant (F_1_ = 6.67, *P* < 0.05). In particular, the percentage of ethanol was 5.26-fold higher in infested as compared to intact fruits. The amount of unidentified volatile compounds in *Penicillium*-infested (7.02%) was similar to that in intact fruits (7.64%). However, when we repeated these analyses considering unidentified volatiles as a sixth compound group, both multivariate and univariate analyses yielded results very similar to those outlined above and, thus, are not detailed here.

We also found overall differences in toughness, pH, and °Brix between intact and infested fruits (F_1, 36_ = 131.55, *P < *0.0001). Specifically, toughness of intact fruits (3.70 ± 0.12 kg) was 4.16 times higher than that of infested fruits (0.89 ± 0.06 kg; F_1_ = 412.90, *P* < 0.0001). The pH of intact fruits (2.47 ± 0.03) was slightly, but significantly, lower than for infested fruits (2.67 ± 0.04; F_1_ = 17.14, *P* < 0.001). Finally, sweetness of intact fruits (15.48 ± 0.25 °Brix) was 12% higher than that of *Penicillium*-infested fruits (13.81 ± 0.16 °Brix; F_1_ = 31.70, *P* < 0.0001).

## Discussion

We chose a novel interaction to experimentally evaluate the effect of fruit infestation by fungi on both fruit appeal to frugivores and vertebrate fruit preferences. Interestingly, and in contrast with most previous work, frugivores consistently preferred infested over intact fruits. Furthermore, the preference for infested fruits was correlated with the higher esters and ethanol levels, but lower sugar levels, in infested as compared to intact fruits^24, 30^. This investigation thus challenges the widely accepted idea of vertebrate avoidance of microbe-infested fruits and reveals a probable mechanism by which microbes facilitate the naturalization of non-native plant species.

Our experimental results show clearly that all three functional groups of frugivores (seed dispersers, pulp feeders, and rodents) preferred fungi-infested over uninfested orange fruits. Such fruit preference took place in very diverse frugivores differing in many traits such as body sizes (e.g. wild boar *vs*. ruddy ground dove in the tropical groves) and feeding habits (e.g. rabbit *vs*. magpie in the Mediterranean groves). Preference for infested fruits was not significant for rodents in the tropical groves. However, our camera traps and field observations indicated that lowland pacas, a frequent rodent visitor, consumed *in situ* infested fruits whereas they usually carried to the jungle uninfested ones. This observation opens the possibility that some of these uninfested fruits were stored^31^ till they become spoiled and their physical and chemical traits altered (see below). The pattern of preference for fungi-infested fruits also held true during the three studied fruiting seasons, supporting the robustness of our findings and suggesting that similar results could be expected for comparable systems.

After Janzen^11^, it has been often reported that frugivores prefer ripe, uninfested fruits over spoiled ones^14^ (see Supplementary Table S1). Nevertheless, changes in fruit preference by frugivores can be expected to vary with vertebrate, plant, and microbe species^14^. Regrettably, most studies reporting preference for intact fruits have relied on feeding trials with captive small frugivorous birds and plant species with small-sized fruits (usually ≤ 1 cm in diameter; Supplementary Table S1). Conversely, apart from the current study, very little is known about the effect of microbe infection on large or medium sized fruits. Fruit size might be consequential for frugivore preferences in relation to infestation due to several causes. Large fruit size allows the possibility of fungal infection altering just the outermost fruit parts (i.e. exocarp) and not the pulp, which may be infected in small-sized fruits. In orange fruits, and probably also in other relatively large fruits, *Penicillium* and other microbes thrive in the exocarp, leaving most of the inner pulp much less infested (Authors *personal observation*). As we revealed, fungal infection softens fruit exocarps, thereby facilitating foraging by some frugivores. We propose that research bias towards small frugivores birds and towards small-sized fruit with thin exocarps have often led to the wrong perception that microbe infection generally lessens vertebrate fruit consumption^19^. Further research on plant-vertebrate-microbe systems is undoubtedly needed. Also, though the infested fruit we offered in our field experiments, exhibited well-developed fungal growth, the question remains as to whether, as the fruit continues to rot, it will become less attractive to frugivores.

Frugivore preference for infested orange fruits opens the non-trivial question of what traits (nutritive, chemical, and physical), caused such pattern of fruit selection^24, 31–33^, despite the lower sugar content of infested compared to control fruits. Many vertebrate frugivores are able to perceive and respond to some odours in the environment through odorant receptors, which are activated by sets of VOCs^24, 25, 34^. In particular, ethanol accumulation in fruits has been long identified as potential signal of high reward^35^ and, in our study, infested orange fruit showed a five-fold increase of accumulated ethanol in the endocarp. Thus, high ethanol concentration of infested orange fruits may explain, in part, frugivore preferences in our tropical and Mediterranean groves^18, 36, 37^.

Other candidate traits are monoterpene hydrocarbons, which were emitted predominantly by intact as compared to infested fruit (69% *vs*. 54%); however, these compounds were partly transformed during *Penicillium* infestation to alcohols and esters. Interestingly, these two chemical classes were further transformed into ethyl esters, which went from representing only 2% of total VOCs in intact fruits to ~20% in infested ones (i.e. a 10-fold increase). Animals so diverse as humans, monkeys, rats and flies are known to perceive and respond positively to esters at very low odour thresholds^30, 38, 39^. Our results thus suggest that esters (within a mixed VOC blend) were perceived as attractive cues by vertebrate frugivores in both tropical and Mediterranean experimental groves. Also the fungus secretes about 50 enzymes involved in plant cell wall degradation^40^, thus softening plant tissues, as indicated by toughness evaluations. This softening probably facilitated frugivore access to fruit pulp, chiefly in the case of small pulp-feeding birds and some rodents unable to penetrate the thick pericarp of intact orange fruits^23, 41, 42^. Finally, we cannot rule out the possibility of frugivore self-medication with *Penicillium*-produced antibiotics, as it has been documented in other similar systems^43^.

Our study supports that vertebrate frugivores, fleshy-fruited plants, and microbes may form a mutualistic ecological triad^44^. In orange fruits, monoterpene hydrocarbons are accumulated at very high levels in oil glands of the exocarp, and these compounds are known to favour fruit colonization by *Penicillium* ^27, 45^. Spoiled fruit and fungus emit VOCs (e.g. esters) that attract frugivore vertebrates which prefer feeding on soft, alcohol- and sugar-rich fruits. Vertebrates frequently disperse viable orange seeds (e.g. wild boars; Authors *unpublished data*) facilitating tree naturalization in tropical habitats^28^ (Authors *personal observation*). Furthermore, recent progress in fungal dispersal has revealed that passage through vertebrate guts can provide a mechanism of dispersal for fungi as well as seeds^46, 47^. Therefore, *Penicillium* and other microbes infecting oranges and other large fruits are likely to benefit from spore dispersal by frugivores. New investigations disentangling the direct and indirect effects likely taking place in these complex multitrophic systems are crucial^23^.

On the other hand, because fungal infection hastens fruit abscission, it has been generally assumed that it has a negative effect on seed dispersal (i.e. fallen fruits are equated to undispersed ones^11, 14^). This is probably the case for most bird-dispersed plants, since birds tend to forage on the canopy and not underneath fruiting trees. Conversely, tree species dispersed to some significant extent by non-arboreal mammals, such as lagomorphs, pigs, other ungulates, and carnivores require fruit dropping to archive seed dispersal, and thus fungal infection also enhances in this way tree dispersal success. Such a cooperative relationships in these cross kingdom interactions is opposed to the more generalized view of the competitive nature of interactions between frugivorous vertebrates and microorganisms^14, 15, 48^ which certainly invites to study in detail other similar systems involving large-fruited plants and contrasting frugivores.

To conclude, we show that frugivores consistently preferred infested over intact orange fruits and that such pattern concords with a high level of attractive volatile compounds in infested fruits. We predict that plant species with large fruits and seeds and dispersed mostly by mammals with acute sense of smell are the most likely to experience enhanced consumption of infested fruits, aided by microbe-induced conspicuous aromas and softened peels. Though some of our target species lack a common evolutionary history, this investigation illustrates a way by which microbes can maintain mutualistic interactions with fleshy-fruited plants and, thus, questioning whether there really is a plant evolutionary dilemma of making their fruits attractive to some frugivores while unattractive to microbes^13, 15, 33^.

## Material and Methods

### Study species

The genus *Citrus* (Rutaceae) comprises several species whose origin is Asian. The orange tree (*Citrus sinensis* L. Osb.) is an evergreen, flowering tree, with an average height of 4 to 8 m. The fruit is a special type of berry named hesperidium, consisting of fleshy parts divided by segments, the whole being surrounded by a separable skin. This is composed of two major regions: the pericarp, commonly known as the peel, and the endocarp, often called the pulp. The pericarp is composed of external coloured peel known as flavedo (exocarp; rich in oil sacs containing volatile compounds), and the internal usually white and spongy layer known as albedo (mesocarp). The inner flesh or pulp consists of segments surrounding the central axis of the fruit enclosed in a locular membrane in which seeds and juice sacs (vesicles formed by highly vacuolated cells containing juice) grow. The acidity of the juice of citrus fruits is largely due to high contents of citric acid, malic acid and fumaric acid, in order of abundance. *Penicillium digitatum* Sacc. causes the most damaging postharvest disease of sweet orange fruits^27^. Dormant *Penicillium* spores present on the fruit’s surface germinate rapidly and colonize injured peel tissue before and during harvesting and processing.

### Study sites

The study was conducted from June 2013 to May 2015 in two very distinct geographical regions, a Mediterranean in eastern Spain and a Tropical in southern Brazil. In both regions we used several experimental groves where frugivore activity was known. In Spain, we selected two Mediterranean sites within the Valencia province in the municipalities of Moncada and Sagunto. In Moncada, we used a 0.6 ha experimental field within the Instituto Valenciano de Investigaciones Agrarias (IVIA; latitude 39°35′N, longitude 0°23′W; 50 m a.s.l.). The Sagunto field site is located near the Sierra Calderona Natural Park (latitude 39°42′N, longitude 0°15′W; 30 m a.s.l), within extensive orange monocultures. Within this site, we used two different orange groves (0.2 and 0.4 ha, respectively) 250 meters apart. The most common frugivore species in these two Mediterranean sites were rabbits (*Oryctolagus cuniculus* L.) and small birds that acted as pulp feeders (i.e. they consume the fruit pulp but without ingesting the seeds^23^). Granivore rodents (e.g. *Mus spretus* L.) and rats (*Rattus rattus* L.) were also relatively frequent visitors. The tropical field site (called Cambuhy; latitude 21°38′S, longitude 48°31′W, 600 m above sea level; 14,083 ha) is located next to a dry tropical forest in Matâo, Sâo Paulo, southern Brazil. Inside the farm there is a large (2,168 ha) semi-deciduous forest of dry Mata Atlantica called Mata da Virgínia. We selected two orange groves (8.1 and 11.5 ha, respectively) about 1.3 km apart of each other. The most frequent frugivore visitors were introduced wild boars (*Sus scrofa* L.) which together with the scarce ring-tailed coati (*Nasua nasua* L.) ingested whole fruits and delivered intact viable seeds (Authors *unpublished data*). Several species of small rodents were also frequent visitors and together with lowland pacas (*Cuniculus paca* Brisson) comprised the guild seed-eating rodents. Pulp feeding bird species such as curl-crested jay (*Cyanocorax cristatellus* Temminck), pale-breasted thrush (*Turdus leucomelas* Vieillot), and ruddy ground dove (*Columbina talpacoti* Temminck) frequently visited and picked the pulp of our experimental fruits. Further detail on the study sites and their frugivore assemblages are documented in Supplementary methods S3 (Supporting Information). All field experiments were done under permission of IVIA, Fundecitrus, and all orange grove owners both in the tropical and the Mediterranean sites.

### Field experimental design

To evaluate vertebrate frugivore preference in the field, intact and *Penicillium*-infested fruit types were simultaneously offered underneath orange trees simulating natural fruit drop on circular sand beds (1-meter of diameter^23^). All frugivores (e.g. mammals, birds) had access to fruit since no exclusion system was implemented^23^. Intact and infested fruits (2 or 3 per type) were alternately arranged, with ~10 cm spacing, beneath each experimental tree. In the experiments of Sagunto and Moncada, 15 fruit depots haphazardly distributed in the field during the months of April to June 2014 and June to December 2013, respectively, coinciding with the ripening seasons. In Brazil, fruits were offered in two parallel rows (10 fruit depots each). Within each row, fruit depots were 60 m apart. Fruit harvesting was recorded during seven and twelve consecutive days in July 2014 and May 2015, respectively. Every one or two days early in the morning the numbers of fruits either consumed *in situ* or removed were recorded and replaced by new fruits of the corresponding treatment. Frugivore identification was based on frugivore tracks on fine sand^23, 49, 50^ and on the way fruits were manipulated and eaten by different frugivores. To confirm the origin of some animal traces and signs, some Bushnell Trophy Cameras with motion sensors were used in Brazil fields. The timing of our experiments was intended to coincide with the ripening of offered fruits.

To inoculate fruit with *P. digitatum*, we used fruits not sprayed with any insecticide and fungicide during at least the previous three months. Fully developed orange fruits were taken to the lab and then their surfaces were disinfested with 1-min immersion in a sodium hypochlorite solution (4 gL^−1^), rinsed with fresh water and left to air dry at room temperature. *P. digitatum* isolated NAV-7 was obtained from the culture collection of the Laboratory of Pathology, Postharvest Technology Center (IVIA, Moncada, Spain). Oranges were inoculated by wounding the rind with a stainless steel tip and introducing 2 µL of a known concentration of 1 × 10^6^ *P. digitatum* NAV-7 spores mL^−1^ in two opposite incisions in the equator of the fruit^45^. Inoculated fruit were placed on closed plastic bags and incubated at 20 °C and 80% relative humidity (RH). Infested fruits were use in the field experiment 7–11 days after inoculation, when the halo of the fungus covered the entire fruit surface (see Fig. 1). A second set of fully developed oranges were used as controls and were treated as infected fruits except that were no wounded and inoculated with *P. digitatum*. We evaluated the possibility that the tiny wounds induce changes on VOC profiles and thus were partly responsible of possible differences between control and *Penicillium*-infested fruits. However, our results unmistakably showed no differences between wounded and unwounded (control) orange fruits for all volatile classes (see Supplementary Figure S2).

### Fruit volatile emissions, physical and chemical traits

**Figure 3:**
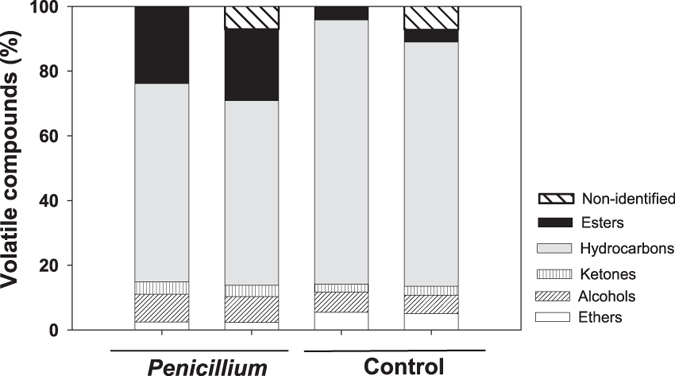
GCMS analysis of the volatile compounds emission in control and *Penicillium-*infested sweet orange (*Citrus sinensis*) fruits. Constituents are classified by chemical class: alcohols, esters, hydrocarbons, ketones, aldehydes, ethers and epoxides. For each treatment (control and *Penicillium*-infested) percentages are shown first without considering non-identified compounds and, then, considering them as an additional class.

To assess how VOC profiles relate to frugivore fruit preferences, chemical analysis of volatiles emitted from intact and *Penicillium*-infested fruits were performed. For *Penicillium*-infested fruits, volatile analyses were conducted in days 7, 8, 9, 10, and 11 after infection, with two fruits per day. The volatile compounds were extracted by headspace solid-phase microextraction (HS-SPME) and analysed by GC-MS essentially as described in Rodríguez *et al*.^45^. Briefly, samples were introduced into glass beakers of 1 L volume (Labbox Labware) closed with foil. 10 μg of 2-octanol (Aldrich, purity ≥99.5%) was added as internal standard. After 1 h of equilibration at room temperature, a 100 μm fiber coated with polydimethylsiloxane (PDMS, Supelco, USA), previously conditioned in the GC injector as indicated by the manufacturer, and was inserted into the glass and exposed for 40 min. The adsorbed volatiles were injected to a gas chromatograph-mass spectrometer (GC-MS) by desorption at 250 °C during 1 min in splitless mode in the injection port of a 6890 N gas chromatograph (Agilent Technologies). Volatile compounds were separated on an Agilent J&W DB-5 ms GC Column (60 m × 0.25 mm × 1.00 μm) coupled to a Termo-DSQ mass spectrometer. The GC interface and MS source temperatures were 260 °C and 230 °C, respectively. Oven programming conditions were 40 °C for 2 min, 5 °C/min ramp until 250 °C, and a final hold at 250 °C for 5 min. Helium was the carrier gas at 1.5 mL/min in the splitless mode. Data was recorded in a 5975B mass spectrometer (Agilent Technologies) in the 35–250 m/z range at 7 scans, with electronic impact ionization at 70 eV. Chromatograms were processed by means of the Enhanced ChemStation E.02.02 software (Agilent Technologies). Compounds in HS-SPME extractions were identified by matching the acquired mass spectra with those stored in the reference library (National Institute of Standards and Technology) and/or by comparison with authentic standard compounds when available. The relative emission rate of every compound in each sample was calculated as its corrected peak area (by fruit weight) divided by the recovery rate of the internal standard. The results are reported as the mean values of peak area percent ± SE. Infested fruits showed comparable VOC profiles independently of the day of sampling after *Penicillium* infection and thus data were pooled. All identified volatile compounds were grouped into five main types (ethers, alcohols, ketones, hydrocarbons, and esters). The results in Fig. 3 are reported as the mean values of peak area percent. For individual volatiles, the results (Supplementary Table S4) are reported as the mean values of peak area ± standard error and the correspondent peak area percent. GC-MS was performed at the Metabolomics Service in Instituto de Biología Molecular y Celular de Plantas, Consejo Superior de Investigaciones Científicas-Universidad Politécnica de Valencia (Spain).

In addition, 20 intact and 20 *Penicillium*-infested orange fruits harvested from 10 trees (2 fruit per tree) were chosen to perform sugar/acid analyses of the juice of each fruit, according to Citrus Handbook (1998). Acidity was measured using a pH-meter (CRISON Basic 207) and sugar content using a refractometer (HANNA HI 96811, expressed in °BRIX ± standard error). Fruit resistance to pressure was measured using a hand-held penetrometer (Penetrometer Fruit Pressure Tester FT011) and expressed in kg as the mean of the peak force at rupture ± standard error following Shmulevich *et al*. (2003). Two measurements were performed on each fruit with the penetrometer.

### Statistical analyses

The results were analysed by fitting generalized linear mixed models using the Proc Glimmix from SAS^51^, which allows the modelling of non-normal response variables as well as the usage of both fixed and random factors^52^. We first evaluated overall frugivore preference for infested *vs*. intact fruits. In this model, the percentage of fruit harvesting was the response variable, whereas fruit infestation and region (tropical, Mediterranean) were specified as fixed factors. Then, we evaluated guild-specific fruit preferences by fitting a second model with frugivore guild (seed dispersers, seed predators, and pulp consumers) and fruit infestation (infested *vs*. intact fruits) as fixed factors. To evaluate the consistence of the effect of one factor at the different levels of other factors, in both models we also included second-order interactions among main effects. When the interaction between any two factors was significant, tests for the effect of a given factor at the different levels of the other factor (i.e. tests of slices) were performed using the SLICE option in the LSMEANS statement of the MIXED procedure^51^. Season (2013, 2014, and 2015) and block (nested within parcel) were included as random factors in both models. To compare the effects of different levels of any significant main factor, we calculated the difference between their least-square means. Because of the binomial nature of the response variables (percentage of fruit harvested), binomial error and logit link function were specified.

Identified VOCs emitted by orange fruits were classified into one of five main groups (ethers, alcohols, ketones, monoterpenes hydrocarbons, and esters). Multivariate analysis of variance (MANOVA) in GLM procedure^51, 52^ were done to test overall differences in the percentages of the five main types of VOCs between *Penicillium*-infested and intact orange fruits. Once overall significant differences were detected, we applied univariate analyses for each group of VOCs. Differences in toughness, pH, and Brix degrees were also tested with multivariate and univariate analyses.

## Electronic supplementary material

Supplementary information
