# Supplementary material for: Fungal infestation boosts fruit aroma and fruit removal by mammals and birds
Source: Sci Rep. 2017 Jul 17;7:5646. doi: 10.1038/s41598-017-05643-z (PMC5514155; doi:10.1038/s41598-017-05643-z)
Supplement: Supplementary file 1 — Supplementary information [file 41598_2017_5643_MOESM1_ESM.docx]

**Fungal infestation boosts fruit aroma and fruit removal by mammals and birds**

Josep E. Peris, Ana Rodríguez, Leandro Peña, and José María Fedriani

| **Frugivore.** | **Frugivore Family** | **Frugivore Order** | **Frugivore length (cm)** | **Feeding on** | **Fruit Family** | **Type Fruit** | **Fruit length** | **Infestation organism** | **Fugivore fruit preference** | **Reference** |
| --- | --- | --- | --- | --- | --- | --- | --- | --- | --- | --- |
|  |  |  |  |  |  |  |  |  |  |  |
| *Bombycilla cedrorum* | *Bombycillidae* | *Passeriformes* | 15-18 | *Ilex opaca* | *Aquifoliaceae* | Small red drupe | 6-12mm | Small darkened or fungal spot | Intact Fruits | Buchholz and Levey 1990 |
|  |  |  |  | *Lonicera maackii maxim* | *Caprofoliaceae* | Bright red to black berry | 2-6mm |  |  |  |
|  |  |  |  | *Prunus caroliniana* | *Rosaceae* | Tiny black cherries | 1cm |  |  |  |
|  |  |  |  | *Phytolacca americana* | *Phytolaceae* | Shiny dark purple berry | 1cm |  |  |  |
|  |  |  |  | *Cornus florida* | *Cornaceae* | [Cluster of two to ten separate drupe](https://en.wikipedia.org/wiki/Drupe) | 10-15mm |  |  |  |
| *Dumetella carolinensis* | *Mimidae* | *Passeriformes* | 22-30 | *Vaccinium corymbosum* | *Ericaceae* | [Blue-black berry](https://en.wikipedia.org/wiki/Berry_(botany)) | 0.7-1.5cm | 14 fruit rot fungi^1^ | Intact Fruits | Cipollini y Styles 1993 |
| *Catharus fuscescens* | *Turdidae* | *Passeriformes* | 16-18 | *Vaccinium vacillans* | *Ericaceae* | Blue to shiny black berry | 1.2cm |  |  |  |
|  |  |  |  | *Gaylussacia frondosa* | *Ericaceae* | Blue, black or white drupe | 1cm |  |  |  |
| *Hylocichla mustelina* | *Turdidae* | *Passeriformes* | 19-21 | *Vaccinium macrocarpon* | *Ericaceae* | Red berry | 9-14mm |  |  |  |
|  |  |  |  | *Arctostaphylos uva-ursi* | *Ericaceae* | Red berry | 7-10mm |  |  |  |
| *Turdus migratorius* | *Turdidae* | *Passeriformes* | 23-28 | *Gaultheria procumbens* | *Ericaceae* | Dry red capsule | 6-9mm |  |  |  |
| *Mimus polyglottos* | *Mimidae* | *Passeriformes* | 25 | *Cornus amomum* | *Cornaceae* | Small blue drupe | 0.6cm | Microbe-infested fruits | Intact Fruits | Borowicz 1988 |
| *Dumetella carolinensis* | *Mimidae* | *Passeriformes* | 22-30 |  |  |  |  |  |  |  |
| *Zonotrichia albicollis* | *Emberizidae* | *Passeriformes* | 17 |  |  |  |  |  |  |  |
| *Poecile hudsonicus* | *Paridae* | *Passeriformes* | 12.5-14.5 | *Cornus canadiensis* | *Cornaceae* | Red drupes | 5mm | Microbes | Intact Fruits | Burger 1987 |
| *Turdus migratorius* | *Turdidae* | *Passeriformes* | 23-28 |  |  |  |  |  |  |  |
| *Poecile atricapillus* | *Paridae* | *Passeriformes* | 12-15 |  |  |  |  |  |  |  |
|  |  |  |  |  |  |  |  |  |  |  |
|  |  |  |  |  |  |  |  |  |  |  |
| *Bombycilla garrulus* | *Bombycillidae* | *Passeriformes* | 32-35.5 | *Sorbus aucuparia* | *Rosaceae* | Small red pomes | 2cm | Fermented fruits | Ethanol preference | Eriksson and Nummi 1983 |
| *Pyrrhula pyrrhula* | *Fringillidae* | *Passeriformes* | 15-17.5 | *Crataegus monogyna* | *Rosaceae* | Oval dark red pome | 1cm |  |  |  |
| *Dasyprocta punctata* | *Dasyproctidae* | *Rodentia* | 42-62 | *Astrocaryum standleyanum* | *Arecaceae* | Ovoid dates | 2.5-6cm | Fermented fruits | Ethanol preference | Dudley 2004 |
|  |  |  |  |  |  |  |  |  |  |  |

**Supplementary Table S1:** Details concerning studies documenting vertebrate frugivore preference for intact or microbes-infested fruits. Note how most available studies concerning the effect of fruit infestation on frugivore preference have focussed on small birds and on small fruited plants. Also, note how previous studies have documented vertebrate vertebrate preference of intact fruits as compared to microbe-infested fruits.

^1^*Alternaria, Aspergillus, Botrytis, Colletotrichum, Cladosporium, Fusarium, Geotrichum, Penicillium, Pestalotiopsis, Phoma, Rhizopus, Saccharomyces, Phomopsis*.

References

Borowicz, V. A. Do vertebrates reject decaying fruit? An experimental test with *Cornus amomum* fruits. *Oikos* **53**, 74-78. (1998).

Buchholz, R. & Levey, D. J. The evolutionary triad of microbes, fruits, and seed dispersers: an experiment in fruit choice by cedar waxwings, *Bombycilla cedrorum*. *Oikos* **59**, 200-204. (1990).

Burger, A. E. Fruiting and frugivory of *Cornus canadensis* in boreal forest in Newfoundland. *Oikos* **49**, 3-10. (1987).

Cipollini, M. L. & Stiles, E. W. Fungi as biotic defense agents of fleshy fruits: Alternative hypotheses, predictions, and evidence. *Am. Nat.* **141**, 663-673. (1993).

Dudley, R. Ethanol, fruit ripening, and the historical origins of human alcoholism in primate frugivory. *Integr. Comp. Biol*. **44**, 315 – 323. (2004).

Eriksson, K. & Nummi, H. Alcohol accumulation from ingested berries and alcohol metabolism in passerine birds. *Ornis Fennica* **60**, 2 – 9. (1983).

**Supplementary Figure S2:** GCMS analysis of the volatile compounds emission of four sweet orange (*Citrus sinensis*) fruits both before (i.e. control) and after being wounded (but not inoculated with *Penicillium*). These four fruits collected in March 2017 from four different trees. Though these fruits showed a somewhat atypical ripening phenology, they were valid samples to evaluate the potential effect of wounding on fruit VOC profile. Volatile compounds were classified as: alcohols, esters, hydrocarbons, ketones, aldehydes, ethers and epoxides. Note how for both values averages across all four orange samples (A) and for individual oranges (B) there were not noticeable differences for any volatile class.

**Supplementary methods and results S3:** Orange tree visitation by frugivores

*1. Methods*

To evaluate vertebrate frugivore preference, intact and *Penicillium*-infested fruit types were simultaneously offered on circular sand beds (1-meter of diameter) underneath tree crowns simulating natural fruit drop (e.g. Fedriani and Delibes, 2013). Frugivore identification was based on frugivore tracks on fine sand (e.g. Fedriani and Delibes, 2013) and on the way fruits were manipulated and eaten by different frugivores. To confirm the origin of some animal traces and signs, some Busnhell Trophy Cameras with motion sensors were used in Brazil fields.

The field experiments were carried out in Mediterranean and tropical orange groves. In the Mediterranean Moncada site, we used a 0.6 ha experimental field within the Instituto Valenciano de Investigaciones Agrarias (IVIA; latitude 39°35’N, longitude 0°23’W; 50m a.s.l.). The most common frugivore species are rabbit (*Oryctolagus cuniculus* L.), black rat (*Rattus rattus* L.), mice (probably Algerian mouse *Mus spretus* Lataste and *Apodemus sylvaticus* L.), common blackbird (*Turdus merula* L.), Eurasian magpie (*Pica pica* L.), house sparrow (*Passer domesticus* L.) and white wagtail (*Motacilla alba* L.). The Sagunto Mediterranean site is located near the Sierra Calderona Natural Park (latitude 39°42’N, longitude 0°15’W; 30 m a.s.l), within extensive orange monocultures. The most common frugivore species in the area are rabbit, garden dormouse (*Eliomys quercinus* L.), black rat, Algerian mouse, house sparrow, common blackbird and European turtle dove (*Streptopelia turtur* L.; Gil-Delgado *et al.*, 2009).

The tropical field site (called Cambuhy; latitude 21°38’S, longitude 48°31’W, 600 m asl) is located next to a dry tropical forest in Matâo, Sâo Paulo, southern Brazil. This is a large (14.083 ha) farm of coffee (*Coffea arabica* L.), orange, corn (*Zea mays* L.) and rubber (*Hevea brasiliensis* (Willd. ex A, Juss.) Mull. Arg.). Inside the farm there is a large (2.168,32 ha) semi-deciduous forest of dry Mata Atlantica called Mata da Virgínia. We selected two orange groves (8.1 and 11.5 ha, respectively) adjacent to the forest and 1.3 Km apart of each other. The main local mammalian frugivores there are ring-tailed coati (*Nasua nasua* L.), wild boar (*Sus scrofa* L.), armadillo (*Dasypus novemcinctus* L., *Euphractus sexcinctus* L. and *Cabassous tatouay* L.), azara’s agouti (*Dasyprocta* *azarae* Lichtenstein), tapeti (*Sylvilagus brasiliensis* L.), lowland paca (*Cuniculus paca* Brisson) and black capuchin monkey (*Cebus nigritus* Goldfuss). The most common frugivore birds are curl-crested jay (*Cyanocorax cristatellus* Temminck)*,* pale-breasted thrush (*Turdus leucomelas* Vieillot), ruddy ground dove (*Columbina talpacoti* Temminck)*,* grey-necked wood rail (*Aramides cajanea* L.), red-eye vireo (*Vireo olivaceus* L.) and rufous-collared sparrow (*Zonotrichia capensis* Muller). Wild boar comes from genetic crosses between European wild boar and domestic pigs (Giménez *et al.*, 2003).

The results concerning frugivore visitation were analyzed by fitting generalized linear mixed models using the Proc Glimmix from SAS (SAS Institute, 2014), which allows the modeling of non-normal response variables as well as the usage of both fixed and random factors (Bolket *et al.*, 2009). We modeled, for each sort of grove (i.e. Mediterranean, tropical) separately, the probability of frugivore visit as a function of consumer guild (seed dispersers, rodents, and pulp feeders). Because of the binomial nature of the response variables (probability of visit), binomial error and logit link function were specified (Bolket *et al.*, 2009).

*2. Results on frugivore visitation*

Frugivore tracks and/or other signs such as feces were found by the fruit in all experimental orange trees and on a large fraction of trials both in the tropical (43.85%; n = 707 night-trees) and Mediterranean (58.60%; n = 884 night-trees) experimental groves. Overall, we recorded 828 frugivore visits to target orange trees (minimum estimate, since occasionally more than one individual could be involved in a single visit). Each visitation was undertaken by one or two frugivore guilds (1.15 ± 0.02 and 1.14 ± 0.01 in the tropical and the Mediterranean groves, respectively). Whereas in the Mediterranean groves we recorded visits by pulp feeders and rodents, in Brazil, in addition to those two frugivore guilds, we also recorded frequent visits by seed dispersers (mostly introduced wild boars).

In the Mediterranean groves, the probability of visit strongly and significantly varied among frugivore guilds (F_2, 2097_ = 53.97, *P* < 0.0001). Specifically, the probability of visit by pulp feeders (0.65±0.16) was 5.9-fold higher as compared with that for seed-eating rodents (0.11± 0.08). In the tropical groves we also found overall significant differences among guilds in their probability of visit (F_2, 2097_ = 53.75, *P* < 0.0001), with pulp feeders being again the most frequent visitors (0.28±0.13), followed by seed dispersers (0.18±0.09), and then by seed-eating rodents (0.05± 0.03).

**Supplementary Table S4:** Volatile terpene compounds identified by GC-MS of *Penicillium digitatum*-infested (A) and control (B) oranges grouped by chemical class, compound, relative percent area and their correspondent standard error.

| **A) Infested fruit volatile emission** | | | | | |
| --- | --- | --- | --- | --- | --- |
| **Chemical class** | **Nº** | **Compound^*^** | **% area** | **Mean area** | **Standard error** |
| **Alcohols** | **1** | Ethanol | 0.13 | 68775114 | 30045196 |
|  | **2** | Isopentyl alcohol | 0.09 | 48556170 | 29688983 |
|  | **3** | 3-Penten-1-ol, 4-methyl | 0.02 | 11179669 | 8851827 |
|  | **4** | 1-Hexanol | 0.08 | 41419066 | 12393808 |
|  | **5** | 1-Heptanol | 0.05 | 28046648 | 7815629 |
|  | **6** | 2-octanol | 1.28 | 698993310 | 349002760 |
|  | **7** | Eucalyptol | 0.07 | 40533186 | 15241533 |
|  | **8** | 1-Octanol | 2.07 | 1132421270 | 305158804 |
|  | **9** | β-Linalool | 1.29 | 704129765 | 208394232 |
|  | **10** | cis-β-Terpineol | 0.07 | 40600870 | 18644233 |
|  | **11** | trans-*p*-Mentha-2, 8-dienol | 0.23 | 124665695 | 73774734 |
|  | **12** | 2-Cyclohexen-1-ol | 0.06 | 32917460 | 20275412 |
|  | **13** | 1-Nonanol | 0.55 | 301495215 | 84344319 |
|  | **14** | α-Terpineol | 1.26 | 687858439 | 200462900 |
|  | **15** | 2-Oxabicyclol [2, 2, 2] octan-6-ol | 0.71 | 388739208 | 75393939 |
|  | **16** | *n*-Tridecyl alcohol | 0.07 | 35576587 | 13416933 |
|  | **17** | 1-Butanol, 2-methyl | 0.03 | 17111691 | 13801211 |
| **Esters** | **18** | Acetic acid, methyl ester | 0.07 | 38888783 | 7713610 |
|  | **19** | Acetic acid, ethyl ester | 2.49 | 1362157384 | 272881095 |
|  | **20** | Propanoic acid, 2-oxo, ethyl ester | 0.08 | 44953622 | 13187921 |
|  | **21** | Acetic acid, propyl ester | 0.04 | 20071972 | 5059717 |
|  | **22** | Acetic acid, isobutyl ester | 0.08 | 44397459 | 11772852 |
|  | **23** | Hexanoic acid, 3-methyl-2-butenyl ester | 2.19 | 1195957102 | 385082615 |
|  | **24** | Acetic acid, 2-pentyl ester | 0.00 | 1790451 | 1000092 |
|  | **25** | Acetic acid, isopentyl ester | 0.23 | 125964727 | 42516352 |
|  | **26** | 1-Butanol, 3-methyl, acetate | 0.07 | 38280113 | 14694910 |
|  | **27** | Acetic acid, pentyl ester | 0.07 | 38096257 | 9240312 |
|  | **28** | Acetic acid prenyl ester | 0.04 | 22780388 | 6708573 |
|  | **29** | Acetic acid, hexyl ester | 2.54 | 1385907728 | 230174101 |
|  | **30** | Acetic acid, heptyl ester | 0.67 | 366754155 | 56990247 |
|  | **31** | 2-Octanol, acetate | 2.22 | 1210988156 | 156312252 |
|  | **32** | Octanoic acid, ethyl ester | 0.12 | 65392475 | 12541945 |
|  | **33** | Acetic acid, octyl ester | 9.22 | 5039849225 | 929405744 |
|  | **34** | Acetic acid, nonyl ester | 1.33 | 724508221 | 131669190 |
|  | **35** | Acetic acid, decyl ester | 0.24 | 129123836 | 27304243 |
| **Hydrocarbons** | **36** | 3-Thujene | 0.01 | 4691267 | 1954280 |
|  | **37** | α-Pinene | 0.22 | 117933393 | 28069804 |
|  | **38** | α-Phellandrene | 0.78 | 424351286 | 162799871 |
|  | **39** | β-myrcene | 0.03 | 17498472 | 6896961 |
|  | **40** | β-Pinene | 0.03 | 15649064 | 6901425 |
|  | **41** | 3-Carene | 0.38 | 206091485 | 54357909 |
|  | **42** | Terpinolene | 0.16 | 86242684 | 20274489 |
|  | **43** | D-limonene | 45.09 | 24650082264 | 6005091974 |
|  | **44** | β-Phellandrene | 0.77 | 420369538 | 129055675 |
|  | **45** | δ-Elemene | 0.47 | 259556140 | 69880261 |
|  | **46** | β-Elemene | 6.16 | 3367554055 | 734103156 |
|  | **47** | β-Caryophyllene | 0.45 | 244204324 | 48206373 |
|  | **48** | β-Cubebene | 0.06 | 33018265 | 11337883 |
|  | **49** | α-Caryophyllene | 0.06 | 33006668 | 11532878 |
|  | **50** | Valencene | 1.67 | 910260434 | 230739460 |
|  | **51** | α-Selinene | 0.55 | 299244022 | 126664886 |
|  | **52** | α-Panansinsen | 0.13 | 73558563 | 14024364 |
|  | **53** | Cyclohexene, 5, 6-diethenyl-1-methyl | 0.41 | 222828236 | 126951112 |
| **Ketones** | **54** | 2-Octanone | 3.41 | 1862238523 | 545997797 |
|  | **55** | *p*-Mentha-1, 8-dien-3-one (+) | 0.12 | 64997105 | 13864282 |
| **Ethers and epoxides** | **56** | Linalool oxide | 0.94 | 516479536 | 178712749 |
|  | **57** | Limonene oxide (*Z*) | 0.60 | 325896335 | 119717026 |
|  | **58** | Limonene epoxide | 0.28 | 153538393 | 80225045 |
|  | **59** | Epoxylinalool | 0.42 | 230952744 | 50481910 |
|  | **60** | Caryophyllene oxide | 0.05 | 27077876 | 9050790 |
| **Non identified** | **61** |  | 0.05 | 26243299 | 11469879 |
|  | **62** |  | 0.07 | 36649388 | 22616062 |
|  | **63** |  | 0.15 | 83921314 | 18252507 |
|  | **64** |  | 0.06 | 33858549 | 7770893 |
|  | **65** |  | 0.21 | 113293842 | 75175554 |
|  | **66** |  | 0.10 | 53953297 | 21419616 |
|  | **67** |  | 0.46 | 252512914 | 61734942 |
|  | **68** |  | 0.20 | 108852073 | 28418960 |
|  | **69** |  | 0.24 | 131164082 | 20492624 |
|  | **70** |  | 0.03 | 16692535 | 6953733 |
|  | **71** |  | 0.05 | 25620158 | 4588383 |
|  | **72** |  | 0.34 | 185653470 | 73542612 |
|  | **73** |  | 2.51 | 1370580891 | 403618689 |
|  | **74** |  | 0.02 | 9822100 | 4013921 |
|  | **75** |  | 0.02 | 12421740 | 5789233 |
|  | **76** |  | 0.76 | 415511459 | 84802331 |
|  | **77** |  | 0.09 | 46924640 | 37258000 |
|  | **78** |  | 0.12 | 63312455 | 21957553 |
|  | **79** |  | 0.04 | 24572441 | 11639679 |
|  | **80** |  | 0.03 | 17870747 | 6737993 |
|  | **81** |  | 0.04 | 21640439 | 13323840 |
|  | **82** |  | 0.09 | 49791874 | 18737342 |
|  | **83** |  | 0.35 | 188648677 | 74855967 |
|  | **84** |  | 0.11 | 59877165 | 18657619 |
|  | **85** |  | 0.11 | 61706739 | 20431045 |
|  | **86** |  | 0.15 | 80929946 | 33176421 |
|  | **87** |  | 0.33 | 177884942 | 38981940 |
|  | **88** |  | 0.19 | 104915805 | 29437529 |
|  | **89** |  | 0.12 | 65240495 | 30563167 |

| **B) Control (healthy) fruit volatile emission** | | | | | |
| --- | --- | --- | --- | --- | --- |
| **Chemical class** | **Nº** | **Compound^*^** | **% area** | **Mean area** | **Standard error** |
| **Alcohols** | **1** | Ethanol | 0.02 | 12176283 | 5262407 |
|  | **2** | 2-octanol | 4.38 | 2230080660 | 284887823 |
|  | **3** | 3-Hexen-1-ol | 1.00 | 508873323 | 137059267 |
|  | **4** | β-Linalool | 0.27 | 139936827 | 40323886 |
|  | **5** | α-Terpineol | 0.07 | 37114326 | 9229008 |
| **Esters** | **6** | Pentanoic acid, ethyl ester | 0.02 | 11681879 | 4844224 |
|  | **7** | Acetic acid, hexyl ester | 0.50 | 253431893 | 46017774 |
|  | **8** | Butanoic acid, ethyl ester | 0.12 | 58800990 | 22790163 |
|  | **9** | Octanoic acid, ethyl ester | 1.27 | 646075995 | 198336980 |
|  | **10** | Nonanoic acid, ethyl ester | 0.04 | 19314823 | 7849216 |
|  | **11** | Butanoic acid, 2-octyl ester | 0.09 | 45473008 | 14722265 |
|  | **12** | 9-Octadecynoic acid, metyl ester | 1.73 | 880230166 | 231255992 |
|  | **13** | 5-methyl-hexanoic acid ethyl ester | 0.06 | 31783913 | 8978856 |
| **Hydrocarbons** | **14** | α-Pinene | 0.04 | 20944566 | 8779958 |
|  | **15** | α-Phellandrene | 0.11 | 54450431 | 9216313 |
|  | **16** | β-Myrcene | 1.34 | 681887061 | 166084502 |
|  | **17** | 3-Carene | 0.07 | 34645597 | 13962043 |
|  | **18** | D-Limonene | 5.47 | 2788835078 | 464274942 |
|  | **19** | Terpinolene | 0.05 | 26988462 | 11278907 |
|  | **20** | Eremophilene | 0.32 | 165235657 | 25344936 |
|  | **21** | β-Elemene | 3.70 | 1883429265 | 435857914 |
|  | **22** | Isocaryophyllene | 0.32 | 161903012 | 60344311 |
|  | **23** | β-Caryophyllene | 24.19 | 12323401625 | 1661686759 |
|  | **24** | β-Cubebene | 0.55 | 279810006 | 72267589 |
|  | **25** | α-Caryophyllene | 2.23 | 1133531350 | 204471777 |
|  | **26** | Selinene | 0.37 | 187568444 | 34005476 |
|  | **27** | Azulene | 1.08 | 547820288 | 104001617 |
|  | **28** | Valencene | 29.55 | 15053505169 | 1814072266 |
|  | **29** | α-Selinene | 2.66 | 1352453010 | 322428678 |
|  | **30** | (-)-α-Panasinsen | 3.43 | 1748366811 | 364050539 |
| **Ketones** | **31** | 2-Octanone | 2.27 | 1158851291 | 173987756 |
| **Ethers and epoxides** | **32** | Limonene epoxide | 0.36 | 185697615 | 48580452 |
|  | **33** | Epoxylinalool | 0.11 | 54952923 | 23567410 |
|  | **34** | Diepicedrene-1-oxide | 0.05 | 27607001 | 7662008 |
|  | **35** | Caryophyllene oxide | 0.64 | 323817924 | 65984395 |
|  | **36** | Calarene epoxide | 0.03 | 13651599 | 4933129 |
|  | **37** | Caryophyllene epoxide | 0.06 | 32994378 | 9335181 |
|  | **38** | α-Cedrene epoxide | 0.09 | 43939556 | 8502018 |
| **Aldehydes** | **39** | Cyclohexane | 3.46 | 1761760013 | 508002713 |
|  | **40** | β-Cyclocitral | 0.20 | 103953892 | 40677234 |
|  | **41** | Longifolene aldehyde | 0.03 | 16644849 | 4413986 |
| **Non identified** | **42** |  | 0.06 | 32712519 | 7131162 |
|  | **43** |  | 0.02 | 8782552 | 3348264 |
|  | **44** |  | 0.05 | 25475204 | 8341801 |
|  | **45** |  | 0.11 | 58321839 | 11494879 |
|  | **46** |  | 0.04 | 19342209 | 5996364 |
|  | **47** |  | 0.02 | 9683733 | 4594622 |
|  | **48** |  | 2.04 | 1040266405 | 202277652 |
|  | **49** |  | 0.07 | 36781243 | 7847909 |
|  | **50** |  | 4.25 | 2165777524 | 497072767 |
|  | **51** |  | 0.82 | 415735295 | 164754063 |
|  | **52** |  | 0.04 | 18601033 | 7881858 |
|  | **53** |  | 0.03 | 13807958 | 6201223 |
|  | **54** |  | 0.02 | 8213705 | 3681012 |
|  | **55** |  | 0.08 | 41816551 | 12777436 |

* The chemical structure of the compounds can be found in Knudsen *et al*. (2006).

REFERENCE

Knudsen, J. T., Eriksson, R., Gershenzon, J. & Ståhl, B. Diversity and distribution of floral scent. *The Bot. Rev.* **72**, 1-120. (2006).
